# Effects of lifestyle modification on metabolic syndrome: a systematic review and meta-analysis

**DOI:** 10.1186/1741-7015-10-138

**Published:** 2012-11-14

**Authors:** Kazue Yamaoka, Toshiro Tango

**Affiliations:** 1 Teikyo University, Graduate School of Public Health, Tokyo, Japan; 2 Center for Medical Statistics, 2-9-6 Higashi Shimbashi, Minato-ku, Tokyo, 105-0021, Japan

**Keywords:** Systematic review, meta-analysis, randomized controlled trial, metabolic syndrome, lifestyle modification

## Abstract

**Background:**

To evaluate the effect of lifestyle modifications on metabolic syndrome (MetS) as assessed by its resolution and improved values for its components.

**Methods:**

This was a systematic review and meta-analysis. Searches were performed of MEDLINE and the Cochrane Database from January 1966 to October 2011 to identify randomized controlled trials (RCTs) related to the study objective. The included studies were RCTs restricted to the English language, with a follow-up period of 6 months or more, which reported overall resolution of MetS or values of MetS components (fasting blood glucose, waist circumference, high-density lipoprotein (HDL), triglycerides, and systolic and diastolic blood pressure (SBP, DBP)). Two investigators independently assessed study eligibility. The effect sizes were the relative proportion of patients with resolved MetS and mean differences in MetS component values from baseline to 1-year follow-up in a lifestyle-modification intervention (LMI) group versus a control (conventional lifestyle education or no treatment) group. Meta-analyses were conducted using a random-effects model.

**Results:**

Eleven interventions in eight RCTs were used for the meta-analyses. The relative proportion of patients with resolved MetS in the intervention group was approximately 2.0 (95% CI 1.5 to 2.7) times greater in the intervention group compared with the control group (7 interventions, n = 2.839). LMI (5 interventions, n = 748) significantly reduced mean values for SBP by -6.4 mmHg (95% CI -9.7 to -3.2), DBP by -3.3 mmHg (95% CI -5.2 to -1.4), triglycerides by -12.0 mg/dl (95% CI -22.2 to -1.7), waist circumference by -2.7 cm (95% CI -4.6 to -0.9), and fasting blood glucose by -11.5 mg/dl (95% CI -22.4 to -0.6) (5 interventions), but reductions were not significant for HDL (1.3 mg/dl; 95% CI -0.6 to 3.1).

**Conclusions:**

The LMI was effective in resolving MetS and reducing the severity of related abnormalities (fasting blood glucose, waist circumference, SBP and DBP, and triglycerides) in subjects with MetS.

## Background

Metabolic syndrome (MetS), also called 'insulin resistance syndrome' [1], 'death quartet' [2], or 'syndrome X' [3], places an individual at risk for type 2 diabetes (T2D) and cardiovascular disease (CVD) [4]. MetS is becoming a worldwide epidemic as a result of the increased prevalence of obesity and a sedentary lifestyle, and the prevalence of MetS in the adult population is relatively high. Four elements comprising MetS have been identified: central obesity, dyslipoproteinemia (increased triglycerides and reduced high-density lipoprotein (HDL) cholesterol), hypertension, and glucose intolerance; however, the definitions used vary somewhat between ethnic groups. Namely, the National Cholesterol Education Program Adult Treatment Panel (ATP) III [5] defined MetS as the presence of three or more of the following conditions: waist circumference greater than 102 cm in men and greater than 88 cm in women (for Japanese, greater than 85 cm in men and greater than 90 cm in women), triglyceride level of at least 150 mg/dl, HDL level less than 40 mg/dl in men and less than 50 mg/dl in women, systolic/diastolic blood pressure (SBP/DBP) 130/85 mm Hg or higher, and fasting blood glucose level 110 mg/dl or higher. The definitions put forward by the European Group for the Study of Insulin Resistance [6] and the World Health Organization [7] are somewhat different from those presented by the ATP III.

The ATP III placed major emphasis on therapeutic lifestyle changes as an essential strategy for clinical management of people at risk for CVD [8]. In an earlier study, we examined the effect of lifestyle modification on patients at high risk of type 2 diabetes and found that a lifestyle modification program was effective in reducing both 2-hour plasma glucose levels and the incidence rate, thus, lifestyle education may be a useful tool in preventing T2D [9]. Changing dietary and exercise behavior may lead to a healthier waistline measurement and body mass index (BMI), improved values for HDL and triglyceride, lower BP, and lower blood glucose. Lifestyle change must be an integral part of risk reduction therapy for MetS and for T2D. However, these recommendations were mainly focused on patients who had specific risk factors for developing MetS, such as obesity, diabetes, high blood pressure, coronary heart disease (CHD), and other risk factors. One reason for this is the variation in the definition of MetS.

The effects of lifestyle modification for patients with MetS are still unknown. To assess the effects of a dietary-based lifestyle modification intervention (LMI; incorporating lifestyle modifications such as 'diet' and 'diet and exercise') on the reduction of MetS and the improvement in values for MetS components in subjects with this syndrome, we conducted a systematic review of randomized controlled trials (RCTs) that examined lifestyle interventions for MetS. We also performed a meta-analysis of the data obtained and conducted several sensitivity analyses. The results of our analyses revealed important information for the treatment of T2D as well.

## Methods

### Study design and search strategy

The study design was a systematic review of the published literature and a meta-analysis of data from each selected study. The study question was whether an LMI program improved the overall resolution of MetS or values of MetS components in adults with MetS, compared with conventional education. A single investigator (KY) searched MEDLINE and the Cochrane Database (data from January 1966 to October 2011) to identify relevant literature restricted to the English language. Free text terms, MeSH terms, and combinations of words were used for search terms. For instance, 'metabolic syndrome', 'exercise', 'physical fitness', 'nutrition', 'diet', 'prevention', 'adults', and 'RCT' were used as search terms (for full details of search strategies, see Additional file 1, Table S1).

### Study selection

RCTs that followed up patients for 6 months or more were included. Randomization of individuals or clusters of individuals were accepted. Reports of lifestyle intervention (combined diet and exercise) or dietary education alone were selected. Specific dietary programs, such as the Mediterranean diet, were treated as dietary interventions. Control interventions were the conventional education usually given to patients with MetS or no treatment. In the systematic review process, if results of one study were reported in more than one publication, we selected the report for the analysis that included the most information at 1 year, and information on the lifestyle and dietary interventions was extracted. Furthermore, if a study reported intervention results for patients with MetS by a stratified analysis, we treated that part of the report as a RCT study. When a trial had 3 or more arms, we treated each arm independently. Two investigators (KY, TT) independently assessed eligibility.

### Data extraction and risk of bias in individual studies

Participants in the examined studies were adults who were diagnosed with MetS according to the ATP III definitions or to other guidelines. To reduce the risks related to MetS, it is necessary that the state of MetS itself be resolved. In selecting studies for this meta-analysis, two main outcome measures from the described intervention were primarily considered. These were the proportion of patients with resolution of MetS and the reduction in the mean values of the major components of MetS (waist circumference, HDL, triglyceride, SBP, DBP, and fasting blood glucose) from baseline to the end of study follow-up (6 months or more; most studies were 1 year). The relative proportion of patients with resolved MetS (RR) was examined primarily to assess the strength of the effects in an LMI group compared with a control group (relative risk was equal to the proportion of patients with MetS in the intervention group divided by the proportion of patients in the control group (RR = Pi/Pc)). If Pc was 0, then 0.5 was added for all the cells in the calculation of RR. The differences in the means of the component measures between the two groups were the other effect sizes of this study. The difference was equal to *d_i _*- *d_c_*, where *d_i _*and d_c _were mean differences from baseline to endpoint in the measurement between the LMI and control groups. When the standard deviation (SD) of the difference from baseline to endpoint was not given in the literature, it was calculated using the following formula:

$$\text{S}{\text{D}}^{2}=\text{S}{{\text{D}}^{2}}_{\text{pre}}+\text{S}{{\text{D}}^{2}}_{\text{post}}\;-2r\text{S}{\text{D}}_{\text{pre}}\text{S}{{\text{D}}_{\text{post}}}_{,}$$

where SD_pre _is the SD of the baseline value, SD_post _is the endpoint value, and *r *is the correlation between the baseline and endpoint groups.

Because no study reported a value for *r*, and its true value was unknown, we consulted our past study data [9] and used *r *= 0.5. Sensitivity analyses were also carried out, using *r *= 0.3 and *r *= 0.7. If the 95% confidence interval (95% CI) was shown instead of SD, SD was calculated using the formula:

$$\text{SD}=(\sqrt{n})\;\left(95\%\;\text{C}{\text{l}}_{\text{upper}}-95\%\text{C}{\text{l}}_{\text{lower}}\right)/4,$$

where *n *denotes the sample size of a group.

Two investigators (KY, TT) independently extracted the data, and disagreements were resolved by consensus. Eight studies were selected for systematic review and meta-analysis, after agreement was reached by both assessors. Studies were mainly assessed for the elements of allocation concealment, blinding, and loss to follow-up, which were listed as an elements of risk of bias table following a Cochrane review [10]. The Preferred Reporting Items for Systematic Reviews and Meta-Analyses (PRISMA) standard [11] was followed during all phases of the design and implementation of the present analysis.

### Statistical analysis

Overall estimates were examined using the random-effects model (DerSimonian-Laird method) [12]), the Bayesian model of Warn *et al*.[13] with non-informative priors, and a fixed-effects model (general variance-based method). A χ^2 ^test was used to assess heterogeneity between trials. A cumulative meta-analysis by the random-effects model was performed to determine at which point sufficient evidence was available to shown a beneficial effect of LMI. Publication bias was visually examined using a funnel plot. The estimates from the random-effects model were plotted. Because 1) the fixed-effects model is useful under homogeneous conditions, 2) the types of LMIs and duration of follow-up durations varied between studies, and 3) the power of statistical tests of homogeneity is low, we planned to use the random-effects model as the primary method, irrespective of the test of heterogeneity. S-plus [14] was used for estimation of the random-effects model and the fixed-effects model, and OpenBUGS [15] was used for the Bayesian model (number of chains = 2, burn-in sample = 10,000, number of Gibbs sampling = 10,000). Significance was set at *P *= 0.05 (two-tailed).

## Results

**Figure 1:**
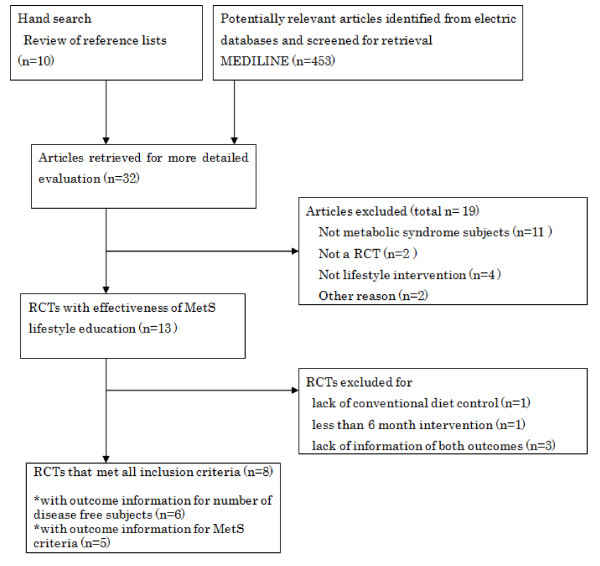
**Systematic review flow diagram**.

The procedure for the systematic review was carried out inn accordance with the PRISMA guidelines [11] (Figure 1). Table 1 summarizes basic information on each trial. Eleven interventions (eight RCTs) in total were assessed [16-23]; one RCT had two types of dietary interventions (that is, different diet programs or 'diet and exercise'), thus each intervention was treated as an independent intervention. Eight interventions (six RCTs) [16-21] were used for the analysis of relative proportion of patients with resolved MetS, and seven interventions (five RCTs) [18,20-23] were used for the analyses of MetS components.

**Table 1:** Characteristics of included randomized clinical trials.^a^

Country, trial	n	Definition of MetS	Age years	Gender	Type of intervention	Duration, years	Rate, % = number free from MetS/number subjects)		Mean difference (SE)					
							
							Intervention	Control	FBG	Waist	SBP	DBP	TG	HDL
UK, CARMEN trial [23]	46	3+ risk factors	46	M/F	LF-SC	0.5	-	-	-	0.0 (2.02)	-13.0 (5.56)	-0.6 (4.29)	50.7 (19.95)	0.5 (2.42)

UK, CARMEN trial [23]	46	≥3 MetS risk factors	46	M/F	LF-CC	0.5	-	-	-	-4.0 (2.94)	-15.0 (6.33)	-4.2 (4.29)	0.0 (16.62)	-1.5 (2.42)

USA [22]	53	Syndrome X	>29	M/F	EX+WL	0.5	-	-	-8.0 (4.84)	-	-6.0 (4.17)	-9.0 (2.08)	-17.0 (64.11)	4.0 (4.26)

Italy [21]^b^	164	ATP III	44	M/F	Med diet	2	20% = 18/90	67% = 60/90	-6.0 (0.35)	-2.0 (0.05)	-3.0 (0.24)	--2.0 (0.15)	-19.0 (0.90)	3.0 (0.24)

Tehran Lipid and Glucose Study [20]^b^	116	ATP III	41	M/F	WR	0.5	0% = 0/40	18% = 7/38	-6.0 (5.18)	0.0 (4.31)	-3.0 (5.58)	-1.0 (3.01)	-15.0 (11.34)	-6.0 (2.22)

Tehran Lipid and Glucose Study [20]^b^	116	ATP III	41	M/F	DASH	0.5	0% = 0/40	34% = 13/38	3.0 (5.83)	-5.0 (4.48)	-11.0 (3.80)	-6.0 (2.96)	-17.0 (11.34)	1.0 (2.16)

Part of DPPRG Study [19]	1711	ATP III	25-82 M/F	M/F	L	3	18% = 107/592	38% = 209/549	-	-	-	-	-	-

Italy [18]^b^	335	ATP III or hs-CRP+2	56	M/F	L	1	34% = 41/120^c^	65% = 77/119^c^	-5.9 (1.28)	-4.6 (0.64)	-6.8 (1.92)	-2.3 (1.03)	-16.0 (8.95)	3.6 (5.84)

Part of PREDIMED study [17]	499	Med diet	67	M/F	Med diet+nuts	3	16% = 41/250	25% = 63/249	-	-	-	-	-	-

Part of PREDIMED study [17]	502	Med diet	67	M/F	Med diet+V00	3	16% = 41/250	21% = 53/252	-	-	-	-	-	-

Part of Finnish DPS study [16]	502	ATPIII high risk	55	M/F	L	1	15% = 26/177	27% = 49/184	-	-	-	-	-	-

ATP III = The National Cholesterol Education Program's Adult Treatment Panel III; CARMEN = Carbohydrate Ratio Management in European National diets; DASH = The Dietary Approaches to Stop Hypertension; DBP = diastolic blood pressure; DPPRG = Diabetes Prevention Program Research Group; DPS = Diabetes Prevention Study; EX+WL = dietary and behavior modification for weight loss plus exercise; FBG = fasting blood glucose; HDL = high-density lipoprotein; hs-CRP+2 = 2 components of metabolic syndrome plus high-sensitivity CRP serum values ≥3 mg/L; L = lifestyle recommendation; LF-CC = low-fat; high-complex-carbohydrate diet; LF-SC = low-fat; high-simple-carbohydrate diet; Med = Mediterranean; MetS = metabolic syndrome; PREDIMED = Prevención con Dieta Mediterránea; SBP = systolic blood pressure; TG = triglycerides; VOO = virgin olive oil; WR = weight-reducing diet; WR+EX = weight-reducing diet plus exercise.

^a^Studies [20-26] were used for the meta-analysis of the metabolic syndrome components.

^b^SD was calculated using 95% confidence interval for references [18,20,21].

^c^Calculated from the proportion

### Types of intervention

The LMIs in the selected studies varied to some degree, and can be divided into those using dietary intervention only, and those using diet plus exercise.

#### Dietary intervention

Several types of dietary education interventions were used in four studies [17,20,21,23], as follows.

The first study used a Mediterranean-style diet with detailed advice about how to increase daily consumption of whole grains, fruits, vegetables, nuts, and olive oil, while the control subjects were required to follow a prudent diet (50% to 60% carbohydrates, 15% to 20% proteins, 30% total fat) [21].

The intervention in the second study consisted of two high-fat Mediterranean diets with virgin olive oil or nuts, with a low-fat diet as the control [17].

In another study, two types of diet were also used as an intervention [20]. One diet was the DASH (Dietary Approaches to Stop Hypertension) diet, which provided 500 kcal less than the subject's daily caloric needs, with increased consumption of fruit, vegetables, and low-fat dairy products, and decreased consumption of saturated fat, total fat, and cholesterol. This diet included more whole grains and fewer refined grains, sweets, and red meat than in usual diet. The other diet was aimed at weight reduction through provision of general information provided both orally and in writing about healthy food choices and recommendations to consume 500 kcal less per day than the caloric needs, based on weight. Control subjects were asked not to make any changes in their normal daily life.

The fourth study used two dietary interventions: a low-fat, complex carbohydrate diet (LF-CC) or a low-fat, simple carbohydrate diet (LF-SC) for 6 months [23]. Control subjects were advised to maintain fat intake at habitual amounts (35% to 40% of energy.)

#### Diet and lifestyle modification

In the remaining four RCTs, dietary education was conducted along with exercise, including exercise [16,18,19,22].

In the first study, lifestyle modification with an individually prescribed diet was given in line with standard guidelines, and advice on exercise was individualized, mainly by suggesting moderate-intensity activity, such as brisk walks for at least 150 minutes/week [16]. Control subjects were provided with general information emphasizing the importance of a healthy lifestyle from their family physicians, who gave advice according to their usual clinical practice.

In the second study, the LMI was to encourage people to make healthy lifestyle choices. Participants in the intervention group were given detailed and individualized dietary and exercise counseling. Sessions for supervised and individually tailored progressive circuit-type resistance training at moderate intensity were recommended twice a week, and offered free of charge by three of the five study centers participating in this research [18]. In addition, because that study reported only the proportions of patients with MetS and those with resolved MetS, the number of patients with resolved MetS was calculated by multiplying the total number of patients and the proportions of resolved and unresolved MetS. Meanwhile, control subjects were given general information about healthy food choices, physical activity, and weight loss at baseline, but with no individualized counseling.

The third of these studies was a three-group randomization trial [19]. In the first group, participants were given an intensive program of lifestyle intervention with the goals of achieving and maintaining a weight reduction of at least 7% of body weight through a healthy low-calorie, low-fat diet, and were instructed to engage in physical activity of moderate intensity, such as brisk walking, for at least 150 minutes per week [19]. Participants in the second group were given standard lifestyle recommendations plus a placebo, and participants in the third group were given standard lifestyle recommendations and metformin. We did not include the third group in the present analysis. Patients were given weekly advice on nutrition for the first 4 weeks, and subsequently every 2 weeks. Caloric intake was restricted using a middle-European balanced diet (~50% carbohydrates, ~30% protein, 20 g to 60 g fat/day supplemented by vitamins and minerals).

Finally, in the fourth study, participants exercised three to four times per week using an identical protocol and participated in a weight-management program in small groups of three to four members [22]. The weight-management program was a behavioral intervention based on a manual, which focused on five elements: lifestyle, exercise, attitudes, relationships, and nutrition. The primary goal of the intervention was a weight loss of 0.5 to 1 kg per week, achieved gradually by decreasing calorie and fat intake through permanent lifestyle changes. Initial dietary goals were set at approximately 1200 kcal for women and 1,500 kcal for men, with about 15% to 20% of calories coming from fat. Control subjects were asked to maintain their usual dietary and exercise habits for 6 months [22].

### Risk of bias

We carried out a summary assessment of risk of bias for the included studies (see Additional file 2, Figure S1). All study designs were RCTs and similar at baseline; however, allocation concealment was unclear in all except in three studies [18,20,21]. Blinding of participants and researcher was also unclear in 6 studies [16,19,22,23] but blinding of outcome assessment was shown in most of the papers. For the outcome value, the number of patients was calculated from the proportions in two studies [17,19]. The response rates of two studies [22,23] were less than 80%, and the proportion of gender varied. Because six of the seven items assessed were unclear in these two studies, the subgroup analysis was conducted excluding these two studies.

### Relative proportion of patients with resolved MetS

**Figure 2:**
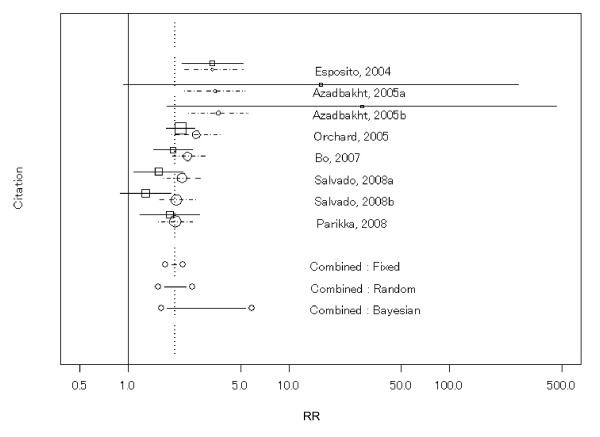
**Forest plot of the relative proportion of patients with resolved metabolic syndrome (MetS) with 95% CI for each study and overall for several models (eight interventions)**. The solid lines extending from the square symbols are 95% CIs of individual studies in the forest plot. Dotted lines extending from the circular symbols are 95% CIs of the cumulative meta-analysis in the forest plot.

Excluding one [18] of the eight studies, the analysis for RR (*n *= 2839) showed evidence of heterogeneity between studies (*P *< 0.001). RR was 2.0 (95% CI 1.5 to 2.7) by the random-effects model for seven interventions, and the other models showed similar values (Table 2). When the analysis was repeated with all eight studies (including the previous excluded study [18]) the results were similar. Figure 2 shows a forest plot for RR with the 95% CIs (shown by lines extending from the quadrangular symbols) of each study and overall estimates by several models (the range of 95% CIs of the overall estimates for several models are shown by a solid line between diamond symbols). All results indicated that the RR was greater in the LMI groups (median of the duration of the follow-up, 2 years) than in the control groups. Because only a few studies were included in the analysis, our planned subgroup analysis was not conducted. A funnel plot showing sample size against effect size suggested little influence from publication bias on the effect size (not shown), but this analysis was severely underpowered, with only eight interventions, and so the conclusions about publication bias were therefore unreliable.

**Table 2:** Comparison of the results of the random-effects meta-analysis (relative proportion of patients with resolved metabolic syndrome (MetS) and difference in mean)

Total (95% CI)	Model	Point Estimate ^d^	95% CI (*lower *to *upper*)
The ratio of proportions patients with resolved MetS (7 interventions)^a^	Random	2.0	1.5 to 2.7
	Bayesian	2.4	1.5 to 5.5
	Fixed	1.9	1.7 to 2.2

Mean difference (7 interventions) ^b^			
Fasting blood glucose, mg/dl	Random ^c^	-11.5	-22.4 to -0.6
(5 interventions)	Bayesian	-5.1	-8.6 to -0.9
	Fixed	-18.7	-20.4 to -17.0
Waist circumference, cm (6 interventions)	Random ^c^	-2.7	-4.6 to -0.9
	Bayesian	-2.6	-4.6 to -0.6
	Fixed	-2.0	-2.1 to -1.9
SBP, mmHg	Random ^c^	-6.4	-9.7 to -3.2
	Bayesian	-7.9	-12.3 to -3.9
	Fixed	-3.1	-3.6 to -2.7
DBP, mmHg	Random ^c^	-3.3	-5.2 to -1.4
	Bayesian	-3.5	-6.2 to -0.97
	Fixed	-2.0	-2.3 to -1.8
Triglyceride, mg/dl	Random ^c^	-12.0	-22.2 to -1.7
	Bayesian	-4.8	-28.1 to 18.8
	Fixed	-18.7	-20.4 to -17.0
HDL, mg/dl	Random ^c^	1.3	-0.6 to 3.1
	Bayesian	0.7	-2.4 to 3.8
	Fixed	2.9	2.5 to 3.3

^a^8 interventions including estimated data denote 2.0 (1.5 to 2.5) for the random-effects, 2.7 (1.6 to 5.8) for the Bayesian, and 1.9 (1.7 to 2.2) for the fixed model, respectively. Number of subjects that could have resolved MetS = 1400 (intervention) and 1439 (control), 2839 in total. Proportions of resolved patients = 35.0% (intervention) and 17.6% (control). ^b^Number of subjects for mean difference = 379 (intervention) and 369 (control), 748 in total. ^c ^Random = DerSimonian-Laird method, 95% CI = 95% confidence interval.

^d ^The estimates by the sensitivity analyses using *r *= 0.3 and *r *= 0.7 were the same with *r *= 0.5 for fasting blood glucose and waist circumference, and it varied for SBP (-7.1, -6.4), DBP (-3.6, -2.9), triglycerides (-12.7, -11.5), and HDL (0.9, 1.6). The estimates by the subgroup analyses using 4 interventions [18,20,21] (random-effects model) denote -3.1 (-5.3 to -0.9) for fasting blood glucose, -3.1 (-5.2 to -1.0) for waist circumference, -5.1 (-8.8 to -1.9) for SBP, -2.3 (-3.3, -1.3) for DBP, -11.5 (-22.4 to -0.6) for triglyceride, and 1.5 (-0.6 to 3.47) for HDL.

### Difference in means for the components of MetS

Overall, the LMI (median 1 year) produced significantly reduced mean values (7 interventions, n = 710) compared with the control intervention as determined by the random-effects model. The values were 11.5 mg/dl (95% CI -22.4 to -0.6) for fasting blood glucose (5 interventions), -2.7 cm (95% CI -4.6 to -0.9) for waist circumference (6 interventions), -6.4 mmHg (95% CI -9.7 to -3.2) for SBP, -3.3 mmHg (95% CI -5.2 to -1.4) for DBP, and -12.0 mg/dl: 95% CI -22.2 to -1.7) for triglycerides. The reduced mean values were not significant for HDL (1.3 mg/dl; 95% CI -0.6 to 3.1). Estimates for the sensitivity analyses for *r *= 0.3 and *r *= 0.7 were not greatly different from that for *r *= 0.5 (Table 2). The results of the subgroup analyses using four interventions denoted similar significance levels.

## Discussion

This meta-analysis provides evidence of the efficacy of an LMI in resolving the proportions of patients with MetS in comparison with conventional education. The proportion of patients with resolved MetS in the intervention group was approximately two times higher in the intervention group than in the control group. Values for five of the six components of MetS (excluding HDL) were significantly reduced in the LMI groups compared with their control groups. Several sensitivity analyses supported these results.

The major goals for subjects in the intensive LMI groups included achieving and maintaining a weight reduction through diet and moderate-intensity physical activity. Most of the control group received only standard lifestyle suggestions. The results of the previous meta-analyses for exercise performed by Carroll and Dudfield [24] were 1.8 mg/dl (95% CI 1.1 to 2.6 mg/dl) for HDL (20 RCTs) and -19 mg/dl (95% CI -26 to -13 mg/dl) for triglycerides (19 RCTs). Because the effect size changes between the study groups were homogeneous, the authors used a fixed-effects model (weighted mean difference method) for the estimation of an overall effect size. The pooled estimate of the effect size was small but significant for both HDL and triglyceride. Our meta-analysis suggested an effect size of 1.3 mg/dl (95% CI -0.6 to 3.1 mg/dl) for HDL and -12 mg/dl (95% CI -22 to -2 mg/dl) for triglyceride by the random-effects model, and 2.9 mg/dl (95% CI 2.5 to 3.3 mg/dl) and -19 mg/dl (95% CI -20 to -17 mg/dl), respectively, by the fixed-effects model. The differences between the two meta-analyses were the type of intervention and study duration. Namely, in our analyses, the LMIs comprised 'exercise and diet' or 'diet only', whereas the in the studies assessed by Carroll and Dudfield, the LMI was 'exercise'. Our meta-analysis required a duration of '6 months or over, median 1 yr', which was longer than the '12 to 52 weeks' required in the meta-analysis by Carroll and Dudfield. Although we cannot deny possible bias, dietary lifestyle intervention is possibly a more effective method than exercise intervention.

A previous meta-analysis of the Mediterranean diet using epidemiological studies [25] found a beneficial effect of the Mediterranean diet on individual components of MetS, including waist circumference, HDL, triglycerides, systolic and DBP, and glucose metabolism. However, it should be noted that in that meta-analysis of the Mediterranean diet, the subjects included high-risk participants. In our analysis, we used only RCTs for patients with MetS, and used several models for analyses, such as sensitivity analyses, because of the heterogeneity of the studies. Nevertheless, our results suggested that the Mediterranean diet and general lifestyle modification, including dietary education, were beneficial.

### Strengths and limitations

To our knowledge, this is the first study employing a meta-analysis of RCT s that has examined the effects of lifestyle modification for individuals with MetS. However, the educational training used was not uniform across the studies. The strengths of our study were that it analyzed only RCTs and assessed the magnitude of the effects primarily according to the relative proportion of patients with resolved MetS, and by the difference in mean changes in individual MetS components. The results suggest that lifestyle modification was more likely than conventional education to result in resolution of MetS. Although we found a weak tendency toward reduction in fasting blood glucose, we consider that fasting blood glucose is not a sensitive index for glucose intolerance. In our experience from a previous study on the effects of lifestyle modification in preventing T2D, a significant effect was shown in the level of plasma glucose 2 hours after an oral glucose load of 75 g, but such an effect was not shown for fasting blood glucose [26]. To confirm whether fasting blood glucose level is a good index as the component for glucose intolerance, further evidence is required.

Certain limitations of this study should be considered. The first is publication bias. From the visual observation of sample size on effect size (figure not shown), the result did not seem to be greatly affected by sample size. We performed electronic searches and a hand search. However, this study used a small number of studies with high heterogeneity, thus a funnel-plot assessment may be not appropriate to examine the publication bias. This analysis was confined to English-language articles, which could also introduce publication bias. However, Moher *et al*.[11] found that language-restricted meta-analyses overestimated the treatment effect by only 2% on average compared with language-inclusive meta-analyses, although the language-inclusive meta-analyses were more precise. Furthermore, we included only RCTs in the present meta-analysis, which could also introduce bias. However, considering that the quality of studies of lifestyle and dietary education may be affected by many confounding biases, these limitations may be acceptable. Publication bias is always a concern in meta-analyses, and although it may be small, we cannot deny the possibility of such bias. We assessed the studies based on the information reported in the papers mainly for the elements of risk of bias table in the relevant Cochrane review [10]. Although the power was low, the significance levels in the subgroup analyses were similar to those of the primary analyses.

A second limitation relates to study duration. We collected data only from studies with a follow-up period extending for more than 6 months. This may be acceptable, however, because earlier assessments could be biased as a result of changes made only because subjects were conscious of being studied. The duration of follow-up in the included studies was 6 months [20,22,23], 1 year [16,18], 2 years [21], and 3 years [17,19]. In the prevention and resolution of MetS, maintaining long-term control is likely to be warranted [27].

A third limitation is the variability in the LMIs. Lifestyle modification was not uniform, and varied depending social circumstances, therefore heterogeneity might be accepted. In most trials, the control diet was the subject's usual diet, whereas the lifestyle modification included some special diets such as the Mediterranean diet [17,21] and DASH diet [20], among others, and most of the studies included recommendations for exercise. Because of this heterogeneity, we used the random-effects model as the primary analysis. Although the quality and content of lifestyle modification varied, the results indicated that it was effective. Because the number of studies was too small to perform difference-by-subgroup analyses, we could not conduct these analyses by intervention styles. Furthermore, even though our search method used systematic review and added hand searching, we could have inadvertently missed eligible studies. The results should be interpreted carefully, considering the risk of bias across studies.

### Implications for practice and research

Many of the changes noted in this review were significant and improvements were observed, but the absolute values were small in terms of magnitude. It should be emphasized that these outcomes measured are surrogate outcomes. Important patient outcomes such as incidence of CHD and T2D should be investigated in future studies.

As the results of the present study show, there is a need for adequate assessment of lifestyle modification for resolving MetS. Future research evaluating lifestyle modification with and without weight loss, lifestyle modification of diet versus aerobic exercise versus combination therapies, lifestyle modification versus medicine, and other comparisons is needed to clarify which aspects and what degree of lifestyle modification are best to resolve MetS.

## Conclusions

This meta-analysis further strengthens the evidence that long-term regular lifestyle modification, including dietary modification only, reduces the prevalence of MetS and abnormalities associated with MetS, and may be a useful tool in reducing the future occurrence of MetS.

## Competing interests

The authors declare that they have no competing interests.

## Authors' contributions

KY and TT designed the study; they had full access to all of the data in the study and take responsibility for the integrity of the data, the accuracy of the data analysis, and the interpretation of the data. KY drafted the manuscript. KY and TT discussed the results and critically revised the manuscript. All authors read and approved the final version of the manuscript.

## Pre-publication history

The pre-publication history for this paper can be accessed here:

http://www.biomedcentral.com/1741-7015/10/138/prepub

## Supplementary Material

Additional file 1 **Table S1**. Search strategy used for MEDLINE. Click here for file

Additional file 2 **Figure S1**. Risk of bias assessment for studies in a Cochrane review. Click here for file
