# Supplementary material for: Effects of lifestyle modification on metabolic syndrome: a systematic review and meta-analysis
Source: BMC Med. 2012 Nov 14;10:138. doi: 10.1186/1741-7015-10-138 (PMC3523078; doi:10.1186/1741-7015-10-138)
Supplement: Additional file 1 — Table S1. Search strategy used for MEDLINE. [file 1741-7015-10-138-S1.DOC]

Additional file 1, Table S1. Search Strategy utilized for MEDLINE

| 1 | Search metabolic syndrome |
| --- | --- |
| 2 | Search "syndrome X" OR "death quartet" |
| 3 | Search #1 or #2 |
| 4 | Search lifestyle[MeSH Terms] |
| 5 | Search "life style" OR "lifestyle"[Text Word] |
| 6 | Search HEALTH-BEHAVIOR[MeSH Terms] |
| 7 | Search HEALTH-PROMOTION[MeSH Terms] |
| 8 | Search HEALTH-EDUCATION[MeSH Terms] |
| 9 | Search EXERCISE-THERAPY[MeSH Terms] |
| 10 | Search PHYSICAL-FITNESS[MeSH Terms] |
| 11 | Search PHYSICAL-EDUCATION-AND-TRAINING[MeSH Terms] |
| 12 | Search behav* OR educ* OR promot*[Title/Abstract] |
| 13 | Search exercise* or physic* activ* or exert* or physic* fit* or sport[Title/Abstract] |
| 14 | Search walk* or jog* or swim* or bicyc* or cycling or weight lift* or gymnastic* or danc*[Title/Abstract] |
| 15 | Search strength or resistance or circuit or penduran*or aerob* or physic* or fit* near train*[Title/Abstract] |
| 16 | Search #9 OR #10 OR #11 OR #12 OR #13 OR #14 OR #15 |
| 17 | Search NUTRITION[MeSH Terms] |
| 18 | Search DIET-THERAPY[MeSH Terms] |
| 19 | Search FEEDING-BEHAVIOR[MeSH Terms] |
| 20 | Search Food Habits[MeSH Terms] |
| 21 | Search nutrit* or diet* or food* or eat*[Title/Abstract] |
| 22 | Search #17 OR #18 OR #19 OR #20 OR #21 |
| 23 | Search #4 OR #5 OR #6 OR #7 OR #8 |
| 24 | Search #16 OR #22 OR #23 |
| 25 | Search #1 AND #3 |
| 26 | Search "randomized clinical trial" OR "clinical trial" OR "controlled trial"[Text Word] |
| 27 | Search randomized[Text Word] |
| 28 | Search #26 AND #27 |
| 29 | Search #25 AND #28 |
| 30 | Search #30 AND (NOT child[Title/Abstract]) |
| 31 | Search NOT child[Title/Abstract] |
| 32 | Search #30 AND #32 |
| 33 | Search #29 AND English[Language] |
