# Supplementary material for: Effects of lifestyle modification on metabolic syndrome: a systematic review and meta-analysis
Source: BMC Med. 2012 Nov 14;10:138. doi: 10.1186/1741-7015-10-138 (PMC3523078; doi:10.1186/1741-7015-10-138)
Supplement: Additional file 2 — Figure S1. Risk of bias assessment for studies in a Cochrane review. [file 1741-7015-10-138-S2.PDF]

UK,CARMEN trial [23]	?	?	?	?	?	+	?
USA [22]	?	?	?	?	?	+	?
Italy [21]	+	+	+	?	+	+	?
Tehran Lipid and Glucose Study [20]	+	+	+	?	+	+	?
Part of DPPRG Study [19]	+	+	+	?	+	+	?
Italy [18]	+	+	?	?	+	+	?
Part of PREDIMED study [17]	?	?	?	?	+	+	?
Part of Finnish DPS study [16]	+	?	?	?	?	+	?
Bias	Random sequence generation (selection bias)	Allocation concealment (selection bias)	Blinding of participants and researchers (performance bias)	Blinding of outcome assessment (detection bias)	Incomplete outcome data (attrition bias)	Selective reporting (reporting bias)	Other bias#

+ Low risk of bias

- High risk of bias

? Unclear risk of bias

Additional file 2, Figure S1- Risk of bias assessment for studies in a Cochrane review
